# The impact of alternative energy technology investment on environment and food security in northern Ethiopia

**DOI:** 10.1038/s41598-022-14521-2

**Published:** 2022-06-21

**Authors:** Daniel Assefa Tofu, Kebede Wolka, Teshale Woldeamanuel

**Affiliations:** 1 School of Natural Resource Management, Ambo University, Ambo, Ethiopia; 2 Wondo Genet College of Forestry and Natural Resource, Hawassa University, Hawassa, Ethiopia

**Keywords:** Environmental sciences, Environmental impact

## Abstract

Energy is a key factor in the economic development. Currently, however, millions of people across the world suffer from energy poverty, having little or no access to energy for cooking, lighting, heating, cooling, or using information and communication technologies. Objective of this study was to investigate the domestic energy sources for households and the impact of biomass use as a source of energy on the environment and food insecurity in the drought-affected northern highlands of Ethiopia. A total of 398 household heads were interviewed using a structured questionnaire, whereas 16 focus group discussions and 12 key informant interviews were conducted. Descriptive data analysis techniques were used to analyze quantitative data while content analysis methods were used to analyze qualitative data. The use of traditional biomass fuels such as firewood, charcoal, crop residue, animal dung, and biomass residue that can be combusted were prevalent in the area, which aggravated the degradation of agricultural lands. As commented by the majority of respondents, the move towards the adoption of modern energy sources was not common due to finance (98%), access (97%), durability (97%) and lack of awareness (93%). The findings showed that land degradation has been severe to the extent that no grain yield can be collected from crop production. As a result, people were exposed to both chronic and transitory food insecurity, and hence the majority of people make their living on food aid. In food-insecure areas, relying on biomass energy could increase land degradation or retard the speed of land restoration, which adversely affects agricultural production and food security. Investing in alternative energy technologies can improve the environment, food security, and people’s health.

## Introduction

Energy is a key factor in socio-economic development^1^. Nevertheless, millions of people across the world suffer from energy poverty, having little or no access to energy for cooking, lighting, heating, cooling, and information and communication technologies. This has a detrimental impact on access to clean water, health services, education, agricultural productivity, and income^2,3^. Access to reliable energy affects the quality of life. Depth of energy poverty is severe, especially in developing countries where the availability of energy is limited or economically unaffordable^4^.

A recent estimate showed that about 14% of the global population lacks access to electricity^5^. Most rural societies in developing countries experience limited access to modern energy services due to problems of either availability or affordability^6^. Due to economic poverty, people rely on traditional fuel sources. A study showed that an increase in household income could promote a transition to modern energy options^7^. In most developed countries and in the few cities of developing countries, the energy comes from expensive sources that are highly polluting and non-sustainable in their nature^8^.

Sub-Saharan Africa is among the regions having the least access to electricity, as more than half of the populations in this region have no access to electricity^9,10^. As the region is less industrialized, about 60% of the available electricity is utilized for domestic purposes, including cooking^11^. Due to the low electric supply, about 86% of the energy demand for domestic use is fulfilled by biomass. In Ethiopia, about 90% of the domestic energy needs have been covered by traditional biomass energy, including the burning of wood materials, charcoal, dung, and crop residues^12^. Despite high potential of hydropower (45GW), only about 15% has been utilized, while less than 1% of the solar, wind, geothermal and biogas energy potentials has been consumed. Since only 5% of the rural population, which is about 79% of the country’s population, could have access to electric power, about 50% of the wood and 30% of agricultural wastes have been utilized for energy^13,14^. In rural areas of low-income countries, the traditional biomass fuel burning technique has been repeatedly reported as inefficient^15–18^. In addition, burning biomass fuels traditionally in indoors could often adversely affect human health due to pollution^6^.

Generally, it is either from traditional biomass or fossil fuels that the energy sector accounts for 65% of total global greenhouse gas (GHG) emissions^19^. Solving the problem related to the energy sector, therefore, is of vital importance to the environment, global society, and the world economy. Switching from traditional biomass to alternative energy sources could be a solution to energy sector and rural poverty problems. The reliance on biomass for fuel leads to deforestation and land degradation, which is the major cause of low crop productivity and results in rural food insecurity. The urgency to shift energy use from traditional sources towards alternative modern sources is not only because of its impact on the environment, economy, and social wellbeing but also because the current capacity of traditional biomass is no longer sufficient to satisfy the fuel demands. The modern or alternative energy sources that attracted special attention include electricity, natural gas, clean cooking fuels, and mechanical power^20^, which are fundamental to supporting the local development by reducing the burden of energy demand on biomass fuel^19^.

Studies reported that many African countries have considerable modern energy potential, which could reduce the burden of rural households resulting from using traditional biomass energy as well as reducing greenhouse gases emissions^20^. According to the African Development Bank^21^, Africa has a large potential of alternative energy, including hydroelectric power (350 GW), wind power (110 GW), geothermal (15 GW) and solar (1000 GW). The solar energy potential, in particular, could be harnessed virtually everywhere in Africa, even though the relative potential could differ with geographic locations. This modern energy source has very little environmental and social impact^8,22^. The continent has a high bioenergy potential, including wood supply from forests, which is estimated at about 520 GWh/year^23^.

Improving access to alternative energy sources for domestic use, particularly in developing regions, is critically essential to meeting the sustainable development goal. This includes increasing access to technologies that make use of traditional fuels in cleaner, safer, and environmentally sound ways. Besides, improving access to modern energy sources such as electricity supply could have multiple benefits for developing regions^5^. For sustainable global development, sufficient modern energy services are critically important^24^, for instance, to reduce the effects of contemporary climate change and socio-economic challenges^22^.

Renewable and sustainable energy expansion needs to increase the availability of energy services to rural areas lacking connections to the grid^25^. In contrast to untouched potential, the move to shift from traditional fuel use to modern renewable energy sources is very limited in Ethiopia. That means the majority of rural inhabitants rely on biomass energy, which could have direct and indirect impacts. In previous studies, little attention was given to the impacts of extensive traditional biomass energy utilization on land degradation and food insecurity. Therefore, the main reasons that triggered this research were: (1) use of biomass for traditional fuel without paying pertinent attention to replacement due to land cover change and land degradation; (2) existence of deep-rooted food insecurity due to land degradation; and thus, many rely on food aid; (3) the traditional cooking stoves are claimed to inefficient and thus, greater biomass is consumed; and (4) limited supply of affordable alternative energy sources. Therefore, the objectives of this study were to assess (i) the rural households relying on biomass energy and their implications on deforestation, land degradation, and food insecurity; (ii) barriers that affect the willingness to adopt modern energy technologies; and (iii) traditional fuel utilization impacts on sustainable livelihood. The study was conducted by involving 398 rural households in northern Ethiopia, where rural people rely on biomass energy and food insecurity has been a challenge for decades due to land degradation and drought.

## Research methodology

### The study area

The study was conducted in the northern highlands of Ethiopia, particularly in the North Wollo and Wag Hemra zones. The North Wollo zone is geographically located between 11°﻿ to 12°N latitude and 39° to 40°E longitude and has an estimated area of 1,275,514.35 hectares, which covers approximately 20% of the region^26^. Weldiya is the zonal capital, which is located 521 km from Addis Ababa. This zone is mostly mountainous and characterized by steep slopes that are hardly suitable for agriculture. Notably, Yassin^27^ shows that 47.3% of the land area is degraded or currently unusable, 24% of the land is arable, 4.6% of the land is pasture, 0.37% of the forest, 17.4% of shrub land, and the remaining 6.3% is comprised of all other uses. The total population of the North Wollo zone is 1,824,361 (50.1% male and 49.9% female)^28^. Lasta district was purposefully selected for this study as it is classified as one of the most food-insecure districts in the zone with a long history of the support of GOs and NGOs via food aid arrangements^29^. Lalibela, known for its monolithic churches, a UNESCO registered heritage, is the capital of the Lasta district.

The Wag Hemra zone is located between 12°15′ − 13°16′ N latitude and 38°20′–39°17′ E longitude, covering a total area of 9039.04 km^2^, and has population of 351,905 (50.1% male and 49.9% female)^28^. Sekota Zuriya district, which is located between 12°23′ and 13°16′ N latitude and 38°44′ and 39°21′ E longitude, was purposefully selected for this study. Sekota town, which is the capital of the district and zone, is 720 km north of Addis Ababa and 540 km north-east of the regional state capital, Bahir Dar^30^.

### Sampling procedures and sample size

Both probability and non-probability sampling procedures were used. First, the North Wollo and Wag Hemera zones were purposively selected based on their reliance on biomass energy, severity of land degradation, and food insecurity. Secondly, the two districts, Lasta from North Wollo and Sekota from the Wag Hemera zone, were also purposefully selected. Thirdly, a total of eight kebeles, which are the smallest level of government administrative unit in Ethiopia, were randomly selected. That means, from each district, four kebeles, namely, Genetemariam, Erfa, Bilibala, and Yimrhane-Kristos from Lasta district; and Wollehi, Abiya, Fikreselam, and Tsemera kebeles from Sekota district, were randomly selected. A total of 398 respondents were randomly selected from 79,058 households using the Yemane^31^ formula *n* = *N/1* + *N* (*e*^2^) at 5% for individual interviews. The household head responded to the structured questionnaire that focused on socio-economic characteristics, domestic energy sources, effects of biomass energy utilization, implications of biomass energy utilization, and factors affecting the adoption of modern energy alternatives.

Besides, participants for focus group discussion (FGD) and key informant interviews (KII) were selected in consultation with development agents. Regarding the FGDs, independent groups of elderly, women, and youth were considered for the guided and open discussion. On the other hand, religion leaders, experts, development agents, and officials at different levels were used for the key informant interviews. The FGDs were held with 8-12 participants and moderated by the researchers using a checklist focusing on the source and use of energy and their associated impacts on land degradation and food insecurity challenges. In general, a total of 16 FGDs and 12 KIIs were conducted. Besides, the district-level offices and departments were visited for secondary data.

A support letter for the study was obtained from Ambo University and informed consent was given by all respondents and discussants.

### Research approach and design

In the study, a mixed research approach was used. The study employed both quantitative and qualitative methods, which enabled the exploration of more complex aspects and relations of the human and social world^31^. Besides, the use of mixed methods has the potential to provide a greater depth of information than is possible by using only qualitative or quantitative methods^32^. The qualitative approach helps to conduct an in-depth study of social and cultural phenomena and focuses on text depending on the observations and descriptions^33^. Moreover, qualitative research is an exploratory type of research and seeks to explain “how” and “why” a particular social phenomenon or program operates as it does in a particular context. As a result, it helps to understand the social environment in which we live and why things are the way they are^34^. The qualitative approach describes and interprets issues or phenomena systematically from the point of view of the individual or the population being studied.

A phenomenological design was used to frame the research and then to explore people’s everyday lives directly related to the environment. Phenomenology is used when the study is about the life experiences of a concept or phenomenon experienced by one or more individuals^35^. In qualitative types of research, the importance of phenomenology is that it attempts to understand how participants make sense of their experiences (it does not assume that participants’ accounts refer to some verifiable reality), but it recognizes that this involves a process of interpretation by the researcher^36^. This is why it is defined as an interpretive process in which the researcher makes an interpretation of the meaning of the lived experiences^37^. To understand the live experience of the individuals and phenomena under study, facts from the individuals involved in the study are gathered using appropriate methods and tools.

### Analysis

In this study, descriptive statistics were used to analyze the quantitative data collected by using the household survey. For this analysis, the Statistical Package for Social Science (SPSS) version 20 and Excel software were used. In order to analyze qualitative data, unpackaged voice data was transcribed and transformed into verbal or text form. Categorization was applied to the transcribed data. To identify themes or patterns (i.e., ideas, concepts, behaviors, and interactions), coherent categories that summarize the whole data set was created after reading and re-reading the transcribed data (text). Categorization is a central step in the analysis that brings together a number of related/similar observations, ideas, and concepts to create meaning for different words and concepts^38^. Finally, content analysis was used as it involves counting the frequency of occurrence of particular words, phrases, or concepts to summarize the whole text data and helps produce a meaningful report^36^.

### Ethics approval and consent to participate

Ethical clearance was obtained from the Research Ethical Review Committee (RERC) of Ambo University, the Director of Research and Community Services, and permission and a supporting letter were obtained from the Amhara zone administration with the facilitation of ORDA (Organization for Rehabilitation and Development in Amhara) before data collection. Verbal informed consent from each participant was obtained during data collection. The respondents were given the right to refuse or to take part in the study. All participants, farmers, and experts were assured of confidentiality. All methods were performed in accordance with the relevant guidelines and regulations of Ethiopia.

### Consent for publish

The authors obtained permission from all participants in the Amhara zone to publish their data.

## Results and discussion

### Livelihood strategies of the community

From focus group discussion and field observation, it was learned that, in the study area, crop-livestock mixed farming is the main means of living. However, crop production takes the highest land share, and this is associated with the limited household livelihood assets. Notably, the limited natural capital, e.g., land shortages coupled with its limited fertility caused by land degradation, is considered a major challenge, which constrains the allocation of land to livestock production. The limited financial capital of households has affected their economic capacity to own animals. Teff, sorghum, and maize are the major crop types cultivated in the area. Although a greater area of land is allocated for crop production, the yield does not satisfy the annual food demand of households, and thus, farmers rely on external food aid provided by GOs and NGOs^39^. In the area, cattle, goats, sheep, donkeys and poultry are commonly reared, although their production and productivity are limited owing to the limited land size, land degradation, and poor livestock management practices.

### Domestic energy sources of the rural household

From the focus group discussion, key informant interviews, and field observation, it was learned that biomass is the major source of energy on which entire rural households rely. All respondents (100%) in the individual interview reported that firewood is used as a fuel for cooking, lighting, and heating purposes. This is even higher than some other Sub-Saharan African countries, e.g., Tanzania^40^, implying high dependence on firewood and pressure on the land. Although the extent of contribution for fuel is lower than for firewood, considerable households use animal dung (85.5%) and crop residue (83.5%) for cooking. About 46% use kerosene, while only 4% use small-scale alternative energy sources like solar for lighting. Besides, very few (4.6%) of the respondents indicated that they use charcoal for heating (Table 1). Crop residue was used, especially in the dry season, after crop harvesting. Poor individuals without access to modern energy sources rely on traditional energy sources, which lead to environmental degradation such as deforestation, soil degradation, and desertification. In Ethiopia, previous studies showed that the share of traditional biomass fuel, i.e., in the form of wood, charcoal, and dung, accounts ~ 90% of the total primary energy use of the household^41^, and about 84% and 99% of urban and rural households, respectively, rely on biomass as their primary fuel for cooking^42^. The practice was also widespread in many Sub-Saharan African countries, such as Zambia, where 97% of rural and 85% of urban households use fuel wood for cooking and heating^43^, and it is the primary source of energy, contributing for more the 70% of the total national energy budget^44^.

**Table 1:** Domestic energy sources in northern highlands of Ethiopia.

Domestic energy sources	North Wollo (n = 215)	Wag Hemera (n = 183)	Average (n = 398)
	%	%	%
Fire wood	100.00	100.00	100.00
Dung cake	84.19	86.79	85.49
Crop residue	85.12	81.97	83.55
Charcoal	3.09	6.05	4.57
Kerosene	47.91	44.26	46.09
Small scale alternative energy (solar or electricity)	3.26	4.92	4.09

The high dependence of rural households on traditional energy sources was mainly associated with the economic development and settlement pattern of the community. Despite the global technological advancement in alternative energy sources, almost all rural households in the study area rely on a traditional energy source: biomass energy. As a result, the use of the remnant forest as the main source of domestic energy has become the question of survival. On one hand, forestland has been declining progressively compared to the situation in the past 30–40 years ago. Ujih et al.^45^ indicated that in the past, fuel wood was simple and the environmental impacts arising from its exploitation were minimal due to the low human population. Following the decline in population and the progressive decline of the forests, however, obtaining fuel wood from the forests in the area is no longer sufficient, and they have started to intensively use crop residue and animal dung as a source of domestic energy.

The discussants explained that cutting trees for firewood had adversely affected land productivity and, ultimately, food security. Due to the previous unwise act, severe degradation has been observed in the area, which led to the loss of livelihood. Although there were attempts to replant and grow trees, it was not as easy as utilizing trees, as the semi-arid environment challenges tree growth. Mazengia^8^ reported that the huge amount of wood extraction, especially in the rural areas, has become a catastrophe to the environment as the extent of tree plantation and replacement is far behind its extraction. Deforestation that has been going on in the area for many decades is a cause of biodiversity loss and soil erosion, which in turn affects the balance of the ecosystem. The growing cumulative effects of using traditional biomass as an energy source on livelihoods necessitate a shift away from traditional energy sources and toward alternative ones.

### Effects of rural household dependence on biomass for fuel on forest resources and land degradation

The key informants and the secondary data indicated that the northern highlands of Ethiopia used to be covered by dense forests. Currently, however, the area is known for its severe degradation. In this regard, historical droughts, consumption of trees for biomass energy, population growth, and the impact of recent climate change were noted as the key reasons for the poor coverage of trees in the area. Discussants mentioned that the communities were severely maltreated by successive droughts of 1955, 1965, 1975, 1985, 1995, 2005, and 2015. Although periods of drought had caused comparable and continued impacts on the community, the 1985 drought caused severe impacts on food security and human lives. The loss of trees and the consequent depletion of natural resources, including fertile topsoil, and consequent poverty, were among the most mentioned.

**Figure 1:**
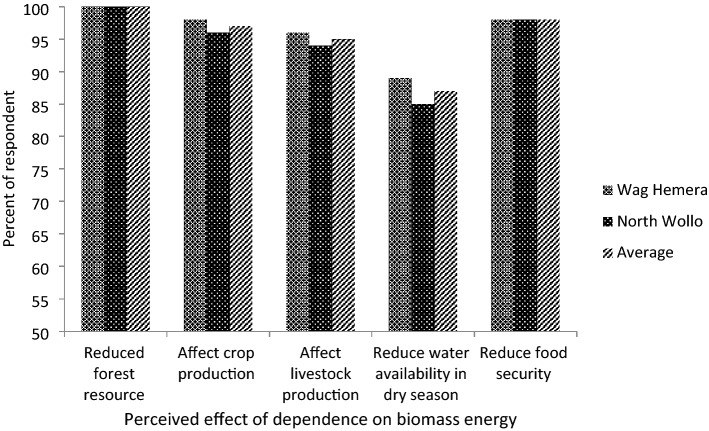
Perceived effects of household dependence on biomass for fuel in Wag Hemera and North Wollo zones, northern Ethiopia.

All the respondents (100%) and discussants underlined that human-induced clearance of trees outweighs the loss of trees due to drought (Fig. 1). A global scale study showed that about 90% of the world’s firewood is utilized by developing countries^46^. An earlier study in Ethiopia estimated the utilization of about 1000 kg of firewood per person per year^47^, which shows the severity of biomass energy utilization on deforestation in areas where all households rely on biomass energy. Although cutting trees for firewood, house construction and other forest products like timber could cause deforestation, conversion of forest land into cultivated land is a dominant cause of deforestation^48^. This has the potential to exacerbate erosion, land degradation, biodiversity loss, food insecurity and poverty^49^. Understanding the multiple effects of deforestation, there is a growing interest in rehabilitating the area through massive plantations by government and non-government organizations. Despite natural and accelerated aridity that hinders the growth of forest biomass, dependence on biomass fuel appeared to be a serious problem for effective rehabilitation in the study area. Globally, long-term reliance on firewood for cooking, heating, and lighting has imposed an additional burden on land rehabilitation and poverty reduction efforts. FAO^49^ indicated that limited availability and access to firewood could exacerbate hunger and poverty by challenging the primary energy source for various purposes, including cooking.

At present, the impact of energy problems extends from being an environmental and economic problem to being a social problem, for instance, imposing women to move a long distance to collect firewood. Mazzoni et al.^24^ reported that traditional fuel utilization had a disproportionate effect on women. It contributed further to the social inequalities that, for instance, caused an uneven distribution of fuel collection, cooking, and childcare responsibilities between men and women within the household**.** Of all these, the worst situation was the limited progress in switching from relying on traditional energy sources to alternative energy sources. The continued dependence on biomass energy could deplete the availability of biomass energy itself due to increasing population and land degradation. In some countries, such as Tanzania, fuel saving stoves have been promoted to reduce fuel consumption, although the transition to modern energy sources is limited^50^. The reliance on biomass energy has also severely affected the forest cover of Uganda^51^.

#### Effects of biomass fuel utilization on agricultural production

Cutting trees for domestic energy purposes, such as for firewood and charcoal, was among the key causes of the continued removal of forest resources and resultant low soil fertility, as 100% of the respondents reported (Fig. 1). The demand for fuel has been increasing due to the ever growing population that relies on biomass for fuel. For cultural reasons, even in urban areas where alternative energy such as electricity is available, households in Ethiopia in general and in the study area in particular still prefer charcoal for cooking and heating. This adds to deforestation and land degradation. Due to severe shortage of wood, animal dung has been used as an energy source for cooking, which should have been utilized for fertilizing cropland. Studies showed that cattle dung contains essential nutrients such as potassium, phosphorus, nitrogen and organic carbon, which have a significant role in soil fertility^52–54^. Thus, utilization of cattle dung for energy could adversely affect crop production.

In the study area, farmers use crop residue, mainly from maize and sorghum, for fuel and residue from teff for feeding livestock. Keeping residue on cropland is recommended from the standpoints of nutrient recycling, soil quality, and erosion reduction^55^. Removing residue, e.g., for fuel, could affect cropland productivity and food security. As a result, the farmers live in an impoverished situation due to land degradation and the presence of both acute and transitory food insecurity. Land degradation’s effect on productivity, including crop and livestock production, had a negative effect on the livelihoods of the community^56^. The biomass production of the area is not sufficient to provide enough feed for livestock. In addition to the energy utilization pattern, the biomass of the study site was highly impacted by the repeatedly occurring severe drought and traditional farming practices.

In both districts, the households could not cover their consumption needs owing to soil degradation, which increased their infertility. About 97% of the respondents relate low productivity to the loss of forest cover, which otherwise could contribute to erosion reduction and climate control (Fig. 1). Deforestation causes drought and flooding, among others, leading to a decline in agricultural yields and food security^57^. The already food-insecure areas have been further affected by the dependence on biomass energy, which could lead to a decline in agricultural production.

About 95% of the respondents reported that the present energy need for firewood has been magnifying the challenges of livestock production and productivity in many dimensions, such as loss of grass, increasing the prevalence of disease, and decreasing access to water (Fig. 1). Land degradation due to forest removal could reduce the availability of forage and water. This meant that farmers faced a significant challenge in finding pasture and water for their animals. The land degradation that is caused by clearing trees or removal of biomass has far-reaching effects. Perceived climate change due to deforestation and land degradation could make pastures less suitable for livestock. For example, temperature affects the environment for livestock production, such as water availability, animal production and reproduction, and animal health, mostly through heat stress^58^. Rojas-Downing et al.^59^ indicated that livestock diseases are mostly induced by increases in temperature and variability in precipitation. Due to various infectious animal diseases caused by climate change, farmers lost their livestock.

About 87% of informants reported that access to water was the most pressing challenge in the area (Fig. 1). Water availability in the area is low, particularly in the dry season, which is associated with increased surface runoff in the rainy season resulting from biomass removal and land degradation due to, partly, removing forest and other biomass for fuel. FAO^60^ stated that forests play a crucial role in the partitioning of water into surface flow, subsurface flow, and evapotranspiration. In contrast, the removal of forest and other biomass strongly impairs the hydrological functioning of the land^61^. Pereira et al.^62^ and Chakravarty et al.^63^ pointed out that any change in land use or land cover can result in significant alterations to the water balance components of a watershed. The observed scarcity of water in the area affected their lives and livelihoods in various ways. Farmers were pressed to travel to distant areas in search of water for their livestock and domestic consumption. It added a double burden on the farmers’ livelihoods through sharing their precious time and by affecting their health and time.

#### Implication of biomass energy utilization on household food insecurity

In the study area, there has been chronic food insecurity for more than three decades^39^. Focus group discussants and 98% of respondents indicated that land degradation partly due to utilization of biomass (fuel wood, charcoal, crop residue, and animal dung) for energy contributed to the existing food insecurity challenges in the area. As a result, farmers’ ability to feed their household from self-production was very low, which is not more than 6 months per year. The remaining months are covered by government and donor-based food aid. For example, over the last five decades, in particular following the Great Ethiopian Famine of 1984–1985, more than 5 million people, the majority of whom are from the northern region, have received food aid in the country on an annual basis, indicating a situation of chronic food insecurity^64^. Land degradation, partly due to biomass energy utilization, has contributed to food insecurity.

The supply of food aid for the poor farmers with the aim of filling the yearly food gap has fallen over two periods. Before 2005, the support of food was offered with the central aim of a humanitarian act. However, since that time, the support has been shaped into developmental forms. That is, the previous provision of food for the needy people in the area was primarily initiated by natural shocks, mainly drought, but the revised forms of support for food aid were aimed at reducing food insecurity through developmental activities such as watershed management practices via the scheme of food-for-work. In the revised approach to food aid, the eligible household, the ”poor-of-the-poor”, could receive aid by participating in developmental activities such as watershed management, which is supposed to restore degraded land and perhaps improve the biomass energy supply. Because of the growing interest in restoring the degraded landscape among governments, NGOs, and development partners, the food-for-work approach has received a lot of attention.

Environmental rehabilitation as a strategy for delivering food relief seemed like a good way to use the relief to develop, but the productivity and quality of outputs were claimed to be generally poor, and the maintenance of soil and water conservation structures and planted trees by them was inadequate^65^. This could be a bottleneck requiring improvement via regular monitoring.

#### Contribution of traditional biomass fuel use for climate change

In the study area, households depend on natural resources for their daily lives. Primarily, to make food, they plough land by converting forest land to agricultural land. Farmers depend on biomass to get the energy they need to fulfill all their domestic energy needs. During this time, they cut trees for firewood and charcoal. Similarly, they also cut trees for house construction and furniture. All of these have already contributed to the emission of greenhouse gases, including carbon dioxide, which is among the major greenhouse gases contributing to global climate change. Fuel wood, roots, agricultural residues, and animal dung are responsible for high emissions of carbon monoxide, hydrocarbons, and particulate matter^17^. It is worthwhile to reduce our domestic carbon dioxide contribution and the resultant climate change by shifting our energy reliance from traditional biomass sources to clean, environmentally friendly, and modern renewable energy technologies. Renewable energy technologies provide an exceptional opportunity for the mitigation of greenhouse gas emissions and to reduce global warming through substituting conventional energy sources^66^. In this regard, unless we act immediately, the cost of the climate impact will be enormous. In addition, the embrace of modern energy forms is indispensable because it is capable of improving the living standards of people, particularly in developing countries, who lack access to services or whose consumption levels are far below those of people in industrialized countries^67^.

### Barriers to adoption of modern energy technologies

The households in the study area faced many challenges that constrained them from adopting alternative energy technologies. Rural households indicated a shortage of capital or finance, access, durability, and awareness as major challenges to adapting to alternative energy technologies, discussed below.

#### Shortage of capital

**Figure 2:**
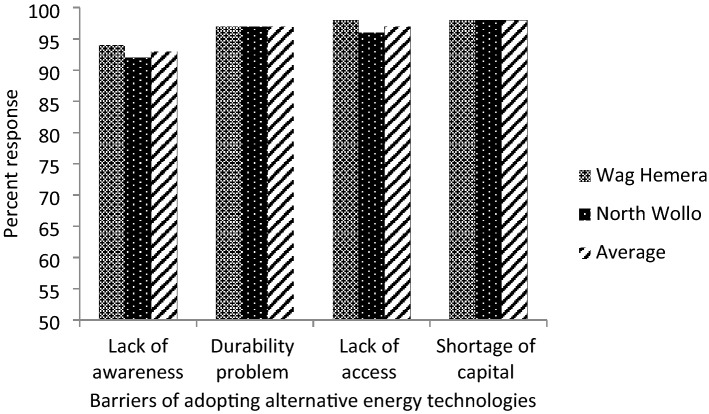
Perceived barriers to adoption of modern energy technologies in Wag Hemera and North Wollo zones, northern Ethiopia.

The majority of respondents (98%) across the study areas reported that a shortage of finance was among the barriers to adopting available modern energy technologies such as solar energy for lighting, radio, and mobile batteries (Fig. 2). This means that different small-scale alternative energy technologies are available on the market, but their adoption is constrained by a lack of finance. For instance, technologies that can be built at the household level like biogas and small solar systems for basic domestic needs such as cooking and lighting, are still difficult due to financial constraints. Quitzow et al.^4^ indicated that for people in Sub-Saharan Africa, energy is less accessible, unreliable, and unaffordable. Sathaye et al.^25^ stated that the high initial cost of renewable energy technologies hinders their large scale adoption, particularly in developing countries, where cost is a prime concern**.** Due to this, the rural people still rely on traditional biomass for their fuel demands, which exposes their lives to environmental, social, and health challenges. For instance, smoke from the use of fuel wood and dung for cooking contributes to acute respiratory infections^57^, which is worse for women in developing countries due to indoor pollution, particularly in houses poorly equipped with living and cooking areas^25,68^.

#### Lack of access to alternative energy source

The results from the FGDs, KII, and household survey (97%) across North Wollo and Wag Hemera zones show that households lack access to alternative energy technologies (Fig. 2). That is, the rural households in the study area have limited access to modern alternative energy technologies for cooking, lighting, and heating purposes. Lack of access to energy, particularly low access to electricity and to other technologies, obliged the community to rely on traditional energy options, which are less efficient and could have adverse effects on household health and put the community at the lower rung of the energy ladder^6,69^. Farmers in the study area have limited energy options and the economic capacity to afford modern energy^70^. This could increase their dependence on biomass energy, which causes deforestation and land degradation, resulting in a vicious circle problem.

Improving households’ access to alternative energy technologies for all residential purposes has diverse benefits. Among the overwhelming benefits, improving the productivity of land and food security could be the major benefit. In addition, modern energy reduces indoor pollution, improves educational outcomes due to light for study, improves health and reduces rural–urban migration^71^. In towns near rural households, where there is better access to the gird electric system, people use electricity mainly for lighting and use wood and charcoal for cooking. In most countries, there is an obvious difference between rural and urban electrification, especially in Sub-Saharan Africa and the South Asian region^72^. Thus, the rural people dominantly rely on traditional biomass energy.

Access to energy is among the key elements for the economic and social developments of Ethiopia^12^. Improved access to renewable modern energy could transform the entire situation and improve the socioeconomic development of the poor. On the other hand, if the experience of using traditional fuel continues with its present pattern, the practice will undermine the strong desire and efforts of the internationally accepted sustainable development goal through the pattern of energy use switch from traditional to new renewable energy consumption. Sustainable development goal #7 seeks to ensure that energy is clean, affordable, available and accessible to all, and this can be achieved with renewable energy sources^73^.

#### Durability problems

About 97% of respondents mentioned that although the rural poor have been trying to adopt some of the small-scale alternative energy technologies, a lack of durability and robustness were the barriers to adopting them even in the face of capital constraints (Fig. 2). Of course, about 4% of the respondents use alternative energy sources such as solar panels (Table 1). Discussants stated that there is a durability problem with alternative energy technologies such as solar. For example, one of the discussant farmers in Wolehi kebele asked a question: “Why are technology producers not reliable or committed to producing durable technology which can work for a long period?”. Currently, although we haven’t adopted much, the technology that we have rarely bought, e.g., solar panels, is not durable. As a result, we lost our scarce money”.

When rural farmers decide to adopt alternative energy technologies by allocating their limited capital, this calls for all those involved in technology innovation to produce and offer technologies that are durable, easy to operate in a rural context, and relatively cheap in price. This may be an opportunity for energy technology producers to get feedback on their products and to contribute their part to protecting the environment and enhancing sustainable development. On the other hand, households or technology users were criticized for their extra concern about the durability, safety, and convenience of new cooking devices. They lack adequate information on the negative health outcomes associated with the inefficient combustion of solid fuels, which has impeded the growth of market demand for clean cooking stoves and other alternative technologies.

#### Lack of awareness

The communities were limited in their awareness of different alternative energy technologies in the market and their contribution to reducing the existing burden on their forest resources. About 93% of informants stated that although there were problems with financial capacity to adopt, having poor awareness about the availability and advantages of technologies affected rate of adoption (Fig. 2). Increasing access to information about alternative energy could improve awareness. Mainstreaming the issue of energy in different development activities is also an option to increase attention and awareness.

### Impact of traditional fuel consumption to sustainable livelihood

In the study area, the limited infrastructure demands huge investments for producing and distributing energy in rural areas, notably to meet the needs for renewable energy options. Three inter-linked objectives, ensuring universal access to modern energy services; doubling the rate of improvement in energy efficiency; and doubling the share of renewable energy in the energy mix, are important to reduce dependence on traditional energy and improve and sustain livelihood^19^. In the move to ensure sustainable livelihood, all the capabilities, assets, and activities required for a means of living are needed to ensure a sustainable situation for human beings to cope with and recover from stress and shocks and maintain their capabilities and assets for the future generations. The high dependence of people on biomass energy for domestic use could affect sustainable livelihoods due to natural resource depletion and limitations on alternative activities, e.g., wood and metal work, pumping ground water for irrigation, that require energy. This implies that all the practice to secure a means of living has seriously affected the productive base for both the current and future generations. This contradicts the widely accepted concept and definition of the World Commission on Environment and Development’s “development that meets the needs of the present without compromising the ability of future generations to meet their own needs”^74,75^.

In the study area, it is evident that deforestation resulted in holistic poverty because it affected all the income sources of the households. Livestock and crop production were seriously affected because of pasture and water scarcity and the decline in soil fertility and rainfall. Taking dramatic action on the approach to shifting from the traditional way of living to modern energy alternatives is critically important. Because optimal use of clean sources of energy could decrease environmental impacts, and improve economic and social needs^73^. Accordingly, the shift from traditional energy use to modern renewable forms of energy sources contributes to reducing the impact on the natural environment, benefits the struggle to ensure food security, and eradicates absolute poverty.

The transition to modern or alternative energy sources should begin with the task of improving rehabilitation efforts and the desire to increase the adaptive capacity of rural households to any natural event, such as a damaging flood. That means the reliance of the rural poor on biomass has adversely affected environmental quality. Thus, investment is needed to reverse land productivity through rehabilitation and reduce dependence on biomass fuel by using alternative energy options. Sawin et al.^22^ noted that **s**ocieties around the world are on the verge of a profound and urgently necessary transformation in the way they produce and use energy^76^. Because they understood that even though it’s convenient to use coal, oil, and natural gas to meet their energy needs, there is a limited supply of these fuels on the Earth and eventually the source will run out since the present rate of use of those nonrenewable sources of energy is much more rapid than they are being created.

## Conclusion

Currently, about 80% of the households in Ethiopia live in rural areas, where traditional biomass is the main source of domestic energy and access to grid-connected electricity systems is uncommon. The traditional energy sources include firewood, charcoal, crop residues, and animal dung. The findings show that all households in the studied area rely on biomass energy owing to the inaccessibility and unaffordability of alternative energy sources. The use of biomass energy has led to negative impacts, that range from environmental degradation (deforestation and land degradation) to food insecurity and health problems associated with smoking and fuel handling. There are many alternative energy sources that could be introduced to the study area, but the opportunity to use them is low. Shortage of capital or finance, limited access to technologies, the durability of the technology, and limited awareness can be mentioned as the bottlenecks to adopting modern energy sources. In food insecure areas, relying on biomass energy could increase land degradation or retard the speed of land restoration, which adversely affects agricultural production and food security. Investing in alternative energy technologies can improve the environment, food security, and people’s health. The present study documented the biophysical and socio-economic effects of extensive dependence on biomass energy based on farmers’ responses. Future studies should quantify the effects based on a reasonable experiment.

## Data Availability

The first author will provide data on reasonable request.

## Abbreviations

BoFED: Amhara Region Bureau of Finance and Economy Development
CSA: Central Statistical Authority of Ethiopia
FAO: Food and Agriculture Organization of the United Nations
FGD: Focus group discussion
GHG: Greenhouse gas
ILRI: International Livestock Research Institute (ILRI)
KII: Key informant interview
PSNP: Productive Safety Net Programme
RERC: Research Ethical Review Committee
ORDA: Organization for Rehabilitation and Development in Amhara
USAID: United States of America for International Development
